# A Ugandan Parenting Programme to Prevent Gender-Based Violence: Description and Formative Evaluation

**DOI:** 10.1177/10497315211056246

**Published:** 2022-01-31

**Authors:** Daniel Wight, Richard Sekiwunga, Carol Namutebi, Flavia Zalwango, Godfrey E. Siu

**Affiliations:** 1 MRC/CSO Social and Public Health Sciences Unit, 3526 University of Glasgow, Glasgow, UK; 2 Child Health and Development Centre, 58588 Makerere University, Kampala, Uganda; 3 47968 MRC/UVRI and LSHTM Uganda Research Unit, Entebbe, Uganda

**Keywords:** parenting, respectability, violence, programme theory, formative evaluation

## Abstract

**Purpose**: To develop a culturally-sensitive intervention for the early prevention of gender-based violence (GBV) in Uganda. **Methods**: Programme design followed the 6SQuID model of intervention development and multi-sectorial advice. A formative evaluation was conducted in two communities with six groups and 138 participants. **Findings**: Four familial predictors of GBV were identified as potentially malleable: poor parent–child attachment, harsh parenting, inequitable gendered socialization and parental conflict. A community-based parenting programme was developed to address them. Its programme theory incorporates Attachment Theory, the concept that positive behavioural control develops emotional control, and Social Learning Theory. Its rationale, structure and content are presented using the TIDieR checklist. A formative evaluation showed the programme to be widely acceptable, culturally appropriate, and perceived to be effective, but also identified challenges. **Conclusion**: The careful development of this community-based parenting programme shows promise for the early prevention of GBV.

In late 2012, the Sexual Violence Research Initiative (SVRI) called for innovative interventions for the early prevention of gender-based violence (GBV). An international workshop in 2013 identified two promising targets for such interventions: Schools and families. Few such interventions existed in sub-Saharan Africa (SSA) and none had been rigorously evaluated and scaled up. To address this gap a team of Ugandan and British researchers planned to develop a parenting programme in Uganda intended to modify familial precursors of GBV and then rigorously evaluate it. To maximise the likeliness of effectiveness, they wanted the programme to be evidence-based and have a clear, logical theory of change. This led them to follow the Six Steps in Quality Intervention Development (6SQuID) model, recently developed by public health professionals in Scotland (Wight et al., 2016).

The 6SQuID model is a pragmatic guide to six essential steps involved in designing an intervention: (1) defining and understanding the problem and its causes; (2) identifying which causal or contextual factors are modifiable; (3) deciding on the mechanisms of change; (4) clarifying how these will be delivered; (5) testing and adapting the intervention; and (6) collecting sufficient evidence of effectiveness to proceed to a rigorous evaluation.

This article describes *Parenting for Respectability* (*PfR*), a community-based parenting programme resulting from the SVRI call. The first version was developed from 2013 to 2014, with input from an Advisory Committee recruited early in 2014, comprising professionals with expertise in GBV from non-government organisations (NGOs), the Ministry of Gender, Labour and Social Development, and Makerere University. The programme then underwent a formative evaluation in three stages from 2014 to 2016.

*Parenting for Respectability* is a 16 session programme for parents and carers of children aged from 0 to 17 years. The article sets out the programme’s rationale, theory, structure and content, and summarises its formative evaluation. It is organized according to steps 1–5 of 6SQuID. The formative evaluation led to several programme modifications, especially being reduced from 21 to 16 sessions. Below, we describe the final version of the programme. The sixth step, a pre-post outcome evaluation from 2017 to 2018, will be reported elsewhere. The TIDieR checklist (Hoffmann et al., 2014) is used to detail the programme.

Throughout ‘parents’ refers to all adults with ongoing caring responsibility for someone under 18 years old, ‘mothers’ to female and ‘fathers’ to male caregivers.

## Step 1: Defining the Problem and Causes

Globally, gender-based violence (GBV), predominantly from intimate partners but also from non-partners, has been experienced by an estimated third of 15–49 year old women in their lifetime. For intimate partner violence, the lifetime prevalence is 27% globally and 33% in the WHO Africa Region. Within this Region, Uganda, a low income country, has almost the highest prevalence estimate at 45% (World Health Organization, 2021). The Uganda Demographic and Health Survey 2016 found that 36% of women had ever experienced partner physical violence, while 22% had ever experienced partner sexual violence (Uganda Bureau of Statistics and ICF, 2018). The potential negative consequences of GBV are well documented, on the mental health and emotional and cognitive development of children, women’s reproductive health, drug abuse and depression (Heise et al., 2002; World Health Organization, 2013), on infectious sexual diseases, especially HIV (Jewkes et al., 2010) and long-term injuries or death (Population Council, 2008), and its effects can span generations (World Health Organization/London School of Hygiene and Tropical Medicine, 2010). GBV is embedded in attitudes that condone violence and traditional practices that perpetuate masculine power, and it thrives where policy, legislation and implementation of laws is weak (Heise et al., 2002; Population Council, 2008).

Gender-based violence and violence against children (VAC) are closely interlinked in several ways (Guedes et al., 2016). They have many shared risk factors, starting in the family, and often occur in the same households. Social norms often support both GBV and VAC, they have similar consequences across the lifespan, and they intersect in adolescence, a time of heightened vulnerability to violence. VAC is extremely widespread globally, with approximately half of all children – one billion aged 2–17 years – reporting having experienced violence in the past year (Hillis et al., 2016). This proportion is similar for Africa, lower for Latin America and Europe, and higher for Asia. The Uganda national VAC survey 2015 found that 59% girls and 68% boys had experienced physical violence in their childhood, and 35% girls and 17% boys had experienced sexual violence in their childhood (MGLSD, 2017). Sexual violence is perpetrated mostly by neighbours and friends, while physical violence is perpetrated mostly by parents and caregivers.

The harmful sequelae of VAC are well documented. They increase with the range of violence experienced and its severity (Felitti et al., 1998; Anda et al., 2010). Immediate consequences are injury and anxiety, although the cultural specificity of these effects is debated (Straus et al., 2013; Lansford et al., 2005), while long-term VAC is associated with the major causes of death in adulthood (Felitti et al., 1998; Anda et al., 2010; Norton & Kobusingye, 2013; Hillis et al., 2000, 2016), often through biological processes (Anda et al., 2010; Danese & McEwen, 2012).

Both GBV and VAC can be transmitted across generations. Parenting practices, especially in the early years, are a key mechanism in the development of persistent antisocial behaviour and violence (Capaldi & Clark, 1998; Farrington, 1998; Gershoff, 2002; Gubi et al., 2020; Hawkins et al., 1998; Herrenkohl & Herrenkohl, 2007; Maas et al., 2008; Murray & Farrington, 2010; Rothbaum & Weisz, 1994; Smith & Stern, 1997). Thus, one important strategy for reducing violence throughout society, including GBV, is to reduce parental violence against children. The evidence for these mechanisms comes overwhelmingly from High Income Countries (HICs) (Lansford & Deater-Deckard, 2012), but increasing research evidence from Low and Middle Income Countries (LMICs) suggests that the associations are the same (Devlin et al., 2018; Fulu et al., 2013; Murray et al., 2013; World Health Organization, 2007).

### Early Prevention of Gender-Based Violence in Low and Middle Income Countries

Since GBV results from factors interacting across all socio-ecological levels (Heise, 2011), preventative interventions operate at many of these and are more effective if targeting several of them (Cork et al., 2018). A systematic review conducted in 2014 (Ellsberg et al., 2015) found promising evidence that in LMICs group training for women and men, community mobilisation interventions, and combined livelihood and training interventions for women can prevent violence. Features of effective programmes included: being participatory; engaging multiple stakeholders; and supporting critical discussion about gender relationships, greater communication and shared decision making among family members, and the development of non-violent behaviour. In a review of randomized trials of interventions tackling intimate partner violence in SSA, Cork et al. (2018) found that physical violence and controlling behaviour seem more amenable to change than sexual violence. Interventions that addressed intimate partner violence as a main aim, and occurred at the community level, had more promising outcomes.

Increasing policy interest in optimising parenting influence provides a great opportunity for the early prevention of GBV. Evidence is emerging that parenting programmes in LMICs to reduce child maltreatment, if delivered by trained facilitators, can improve child outcomes (Engle et al., 2011; Knerr et al., 2013; Lachman et al., 2018, 2020; Shenderovich et al., 2019; Singla et al., 2015). Given the shared risk factors for VAC and GBV, such programmes may also reduce subsequent GBV. However, a scoping review found only two interventions directly intended for the early prevention of both IPV and VAC in LMICs, both of them targeting fathers (Bacchus et al., 2017). *REAL Fathers* in northern Uganda (Ashburn et al., 2016) and the *One Man Can Fatherhood Programme* in South Africa (Hatcher et al., 2014) were both delivered in community settings with group education and discussion sessions, although *REAL Fathers* supplemented this with individual and couple mentoring sessions. Although male only interventions are far more successful at recruiting fathers than parenting interventions aimed at both sexes, there is growing recognition that fatherhood programmes need to be accompanied by initiatives to support women exposed to family violence (Bacchus et al., 2017).

In Uganda, there are various NGO community-level initiatives to tackle GBV (e.g. Abramsky et al., 2016; Ashburn et al., 2017) and in 2020 the government endorsed the Spotlight Initiative, a United Nations and European Union programme to eliminate all forms of sexual and gender-based violence, primarily through women’s economic empowerment. However, while various legislation outlaws such violence, it is perpetuated through widespread social norms (Ashburn et al., 2017) and no early prevention has been scaled up.

## Step 2: Identifying Modifiable Causal Factors

Reviews of the precursors of GBV identify four key, interrelated factors perpetuated within families: Poor child attachment and parental bonding, harsh parenting, inequitable gendered socialization and parental conflict (Harvey et al., 2007; Jewkes, 2002; Population Council, 2008). The first two are of fundamental importance to optimizing child development and wellbeing, while the last two are more specific to GBV prevention.

### Poor Parental Bonding and Child Attachment

Poor parent–child relationships can start with difficulty bonding with a newborn baby (Butchart et al., 2006; Djeddah et al., 2000) and are a critical risk factor for maltreatment (Brown et al., 1998; Trentacosta et al., 2008). According to Attachment Theory – which drew on research in Uganda (Ainsworth, 1967) – secure attachment and parents’ responsiveness to their child are critical for the child to develop confidence, emotional regulation, empathy and cognitive ability (Ainsworth et al., 1978; Belsky & Fearon, 2008; Bowlby, 1982). Conversely, parental maltreatment commonly results in ‘disorganized’ insecure attachment, with infants unable to deal with difficulties. They either become punitively controlling when older or remain very disorganized (Main & Solomon, 1990) and are particularly likely to perpetrate VAC and/or GBV (Dutton, 1999; Schwartz et al., 2006).

### Child Maltreatment

As noted above, much evidence associates child maltreatment with later interpersonal and GBV. It is deeply rooted in cultural and economic practices and is transmitted across generations (Glaser, 2000). In Uganda, as in much of SSA, policy and practice on corporal punishment do not match. Although abolished in schools, it is almost universally accepted (Devries et al., 2014), and surveys find it widespread in the home and in schools (Global Initiative to End All Corporal Punishment of Children, 2017). Child maltreatment is less likely with positive parent–child relationships (Bostrom, 2003; Bronfenbrenner, 1979; Felitti et al., 1998; McKee et al., 2007), high levels of paternal care, and awareness of child development (Bavolek, 1989; Butchart et al., 2006).

### Inequitable Gendered Socialisation

Of these four factors, there has been least research on inequitable gendered socialization. However, it establishes gender roles and expectations that reproduce and legitimate systematic gender inequalities in rights, resources, opportunities and protections, justifying GBV (Wood et al., 2008), and is generally more pronounced in LICs, rural areas, and with lower education. It involves norms that, typically, encourage girls to be accommodating and deferential and boys to be strong and assertive, often leading to differential access to, for instance, nutrition, health care and education (Connell, 1987). Evidence from Uganda suggests that inspirational mothers and gender-sensitive, supportive fathers can improve their daughters’ self-confidence, motivation and ambition (Warrington, 2013).

### Parental Conflict

Witnessing intimate partner violence damages children’s psychological wellbeing (Herrenkohl & Herrenkohl, 2007; Lichter & McCloskey, 2004) and makes them more likely to become violent (World Health Organization, 2006). Boys are at risk of becoming perpetrators of GBV as adults (Abrahams & Jewkes, 2005) and girls victims (Gubi et al., 2020; Seedat et al., 2009). A strong relationship with a caring adult, usually the mother, can mitigate against these impacts (Holt et al., 2008).

### Programme Aims

The project’s Advisory Committee resolved that these factors might be malleable, given evidence of historical change and the effectiveness of some parenting programmes and of relationship counselling and mediation. It was decided that the programme should aim to reduce child maltreatment and prevent GBV by modifying these four familial precursors of GBV.

## Step 3: Mechanisms of Change

The next step was to identify the mechanisms by which these familial factors might be modified, drawing on the team’s prior understandings of behaviour change. A community development social worker, CN, was recruited to develop the programme and subsequently facilitate it. Having drafted learning outcomes for 21 sessions we identified exercises that already met these outcomes from existing programmes; new exercises had to be developed on communication and spousal relationships, led by CN. The team’s strategic approach followed four broad principles, and the theory of change incorporated three specific theories.

### Strategy

The programme incorporates four generic principles, but within pragmatic limits. The first is to harness existing motivation, specifically to maximise respectability. Interventions are far more likely to be effective if they harness existing motivations (Michie et al., 2011), are ‘culturally compelling’ (Panter-Brick et al., 2006) and sensitive to the experiences of those targeted (Morelli et al., 2018). A key concern of parents in East Africa, and probably across SSA, is the family’s respectability (*heshima* in Swahili, *ekitiibwa* in Luganda), requiring children’s good behaviour and respect for their parents and other adults (Siu et al., 2013; Wamoyi & Wight, 2014). Many parents believe that numerous contemporary influences undermine this, in particular schooling, the media, Children’s Rights and restrictions on corporal punishment. *PfR* tries to address these concerns without reproducing undesirable norms.

The second is to work with participants’ existing experience, understanding and skills. The ICDP’s approach is to ‘Start with what they know, build with what they have’ (*Lao Tsu 700 B.C*), identifying indigenous child-rearing practices to develop, rather than impose external concepts and restrictions. This is done through facilitative, rather than instructive, guidance, encouraging active involvement and sharing. Such community empowerment develops participants’ confidence to change their circumstances through ‘the problem-posing, problem-solving process’ (Bitel, 2012: 1). This method was partially incorporated into *PfR* activities, especially on bonding and attachment, with input from a Tanzanian ICDP facilitator. However, the programme’s explicit objectives and use of existing materials from evidence-based programmes prevented communities truly identifying their own solutions.

This meant the development of *PfR* was only partially participatory, the third principle, which is the reality for most ‘participatory’ projects (Cornwall, 2009). *PfR*’s content was informed by previous research on parents’ concerns (e.g. Siu et al., 2013; Wamoyi & Wight, 2014), it involves the mobilisation of community leaders, and it evolved through the initial participants’ and facilitators’ input. However, the programme’s main goals were established a priori by the research team and, following revisions arising from the formative evaluation, it will not be adapted again to meet target communities’ self-defined priorities. It therefore risks being perceived as an external imposition (Bunton et al., 1991).

The fourth principle is to intervene at different levels in the socio-ecological framework (McLeroy et al., 1988). *PfR* is intended to operate at three different levels: the intrapersonal, through modifying knowledge and attitudes; the interpersonal, in particular couple and family relationships; and the community, through involving formal and informal community leaders, participants’ families and neighbours in homework exercises, and sharing learning through community events, thus changing community norms. We also anticipate it will be complemented at the policy level by initiatives from Ugandan NGOs and government.

### Programme Theory

#### Underlying Theories

Three specific theories underlie the intended mechanisms to modify family practices. All operate at the intrapersonal and interpersonal levels, while the third, modelling, also operates at the community level. The first is Attachment Theory, outlined above. This programme aims to help parents become more aware of, and sensitive to, their child’s behaviour and psycho-social needs (cf. Cooper et al., 2009), and respond consistently with warmth, acceptance and support, which should strengthen the child’s trust, security and self-worth. The programme should also encourage parents to communicate with their child about her/his experiences and about others’ feelings, promoting both attachment security and empathy.

The second theory is that parents’ positive behavioural control can promote their child’s emotional control, cooperation and affection for their parents (Hutchings & Gardner, 2012; Gardner, 1989; Grusec & Goodnow, 1994; Webster-Stratton, 1998). Positive interactions, including shared activities, praise, and child-directed play, are likely to foster child cooperation and self-worth. This should have the opposite effects of harsh and/or inconsistent parenting, but it requires more time, especially important in the context of infinite domestic work. However, these strategies should not disempower parents but lead children to be more admiring of, and caring for, them.

Third, Social Learning Theory argues that new behaviours can be learnt, and existing behaviours changed, through modelling behaviour, either by demonstration or being taught (Bandura, 1986). ‘Modelling’ involves the development of self-efficacy, intentions and planning, and modifying social approval (Bandura, 1986). People observe credible role models, with whom they can identify, engaging in particular behaviours, they see the benefits of these behaviours, are motivated to adopt them, and this is then positively reinforced by significant others.

#### Causal Pathways

**Figure 1.**
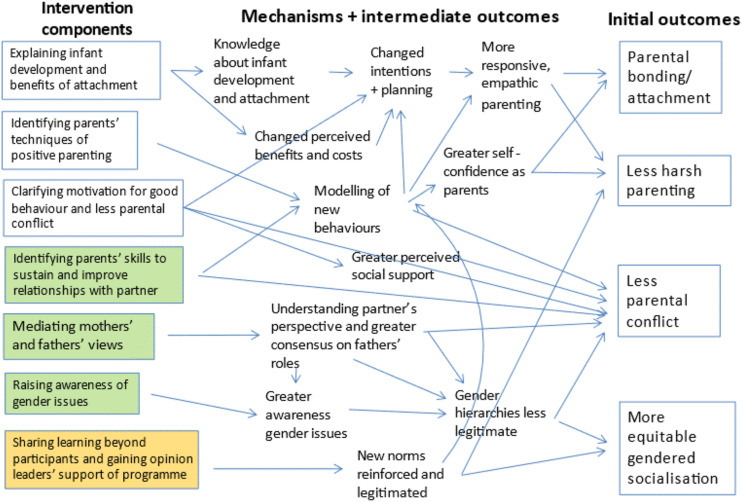
Intended causal chains between intervention components and initial outcomes.

**Figure 2.**
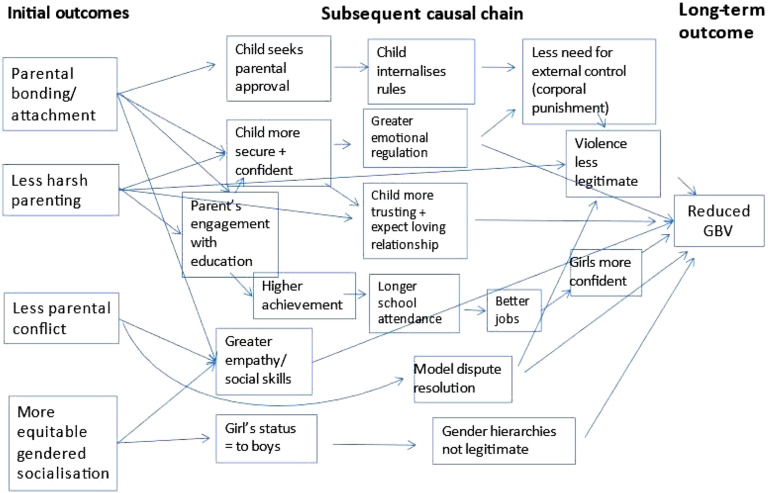
Intended causal chains between initial outcomes and reduced GBV.

The main causal pathways by which the programme is intended to work can be divided into two stages: How the principle intervention components are intended to modify the four elements of family life (Figure 1) and how their modification will lead to reduced GBV (Figure 2). However, to avoid excessive complexity these diagrams are not comprehensive. The first three components (unshaded) are about parent–child relationships, the next three (green) about inter-partner relationships and gendered socialization, and the last (yellow) covers how the programme is intended to operate at the community level.

Figure 2 shows how improved parental bonding and child attachment should help reduce GBV in at least five ways: By leading children to internalise rules, have greater emotional regulation, expect loving relationships, have greater social skills and by increasing parents’ engagement with their children’s education. Less harsh parenting should contribute to at least three of these pathways. Reduced parental conflict should help children develop empathy and social skills and provide constructive models for resolving disputes, while more equitable gendered socialisation should facilitate empathy and social skills and help establish a norm of gender equity from childhood. Given the length of these intended causal chains, they are much harder to test empirically than those in Figure 1.

### Programme Structure and Content: Template for Intervention Description and Replication

Here, we describe *PfR* as revised after the formative evaluation. The content is delivered through 16 weekly sessions, each lasting approximately 2 hours. Inspired by the HIV programme *Stepping Stones*, the first nine sessions are single-sex, allowing mothers and fathers to explore issues separately. Single-sex groups are then merged and divided into two mixed-sex groups for the remaining sessions, in which different gendered perspectives can be expressed and, ideally, resolved. During recruitment this allows the programme to be presented to men as ‘a fathers’ programme’, rather than a generic ‘parenting programme’ which is widely understood to be primarily for mothers (Siu et al., 2017). Table 1 describes the intervention according to the TIDieR checklist (Hoffmann et al., 2014) and Table 2 describes the content of the 16 sessions.

**Table 1.** Parenting for Respectability: TIDieR Checklist.

Item	Description
1. Name	Parenting for Respectability
2. Rationale and theory	Four features of family relationships have been associated with GBV: Poor child attachment and parental bonding; harsh parenting; inequitable gendered socialization and parental conflict. *PfR* aims to modify these for the early prevention of GBV. The programme’s underlying theories are Attachment Theory, the concept that positive behavioural control develops emotional control, and Social Learning Theory
3. Materials	See Table 2. Exercises drawn from *Program H* (www.promundoglobal.org), *Stepping Stones* Welbourn et al. (2016), *Mema kwa Jamii* Wight & Remes (2010), the *International Child Development Programme* (www.ICDP.info) and *Sinovuyo Caring Families Programme (or Parenting for Lifelong Health for Young Children)* Lachman et al. (2018) and developed de novo
4. Procedures	Each session aims to raise awareness of the issues; draw on existing skills and experiences of participants through appreciative enquiry; and develop parenting skills and practices. They use reflection, games, small group work, pictures and role-playing practical skills. Most conclude with home exercises to practice learning during the following week, thus sharing it with relatives and neighbours, and most start by reviewing this home practice
5. Who provides	Sessions are delivered by two facilitators selected from each group and then trained for 5 days by a community development professional. Approximately 20% sessions supervised by trainer
6. Mode of delivery (step 4 6SQuID)	See Table 3. Delivered in group sessions of approximately 20 participants. The first nine sessions are single-sex, groups are then merged and divided into two mixed-sex groups for the remaining sessions
7. Where	Villages/wards with no existing/recent programmes tackling VAC or GBV, or programmes involving substantial material incentives. Programme delivered in local venues identified by participants
8. When and how much	Delivered in 16 weekly 2 h sessions. Participants choose optimal day and time
9. Tailoring	Sessions adapted to participants’ experiences and understanding (see 4 above). Some participants sought individual advice from facilitators on marital relations
10. Modifications	Many sessions overran to 3 h. Some were postponed by a week. In many sessions, especially in Kigungu, the trainer, CN, co-facilitated with the lay facilitators. In Kigungu, an original facilitator was replaced. Revisions following formative evaluation are summarized above (Development of programme)
11. Assessing fidelity	In formative evaluation all sessions supervised by trainer, CN, and 20 sessions in each of two communities observed by RS (see below)
12. Actual fidelity	Confidence and competence of facilitators varied considerably. Some exercises only partially implemented and/or engaged in. CN co-delivered most of programme in Kigungu; in Bweya the facilitators delivered most

**Table 2.** Parenting for Respectability Sessions.

	Content	Main Goal
Single-sex		
1	Importance of parent–child interactions from birth; effects on child’s wellbeing	Parent–child connectedness
2	Beliefs and practices regarding parent–child interactions. Value of positive interaction	Parent–child connectedness
3	Child development: Pre-birth to primary school. Importance of father’s support during pregnancy	Parent–child connectedness
4	Influence of one’s own parents. Identifying and learning positive discipline techniques	Parent–child connectedness
5	Values, beliefs and practices in child rearing and ‘respectability’; relationship to children’s behaviour	Reduce harsh parenting
6	Harm of gender norms to both women and men. Power in relationships and impact on individuals and relationships	Gendered socialization
		Inter-partner relationships
7	Own experiences of partner conflict. Causes of conflict	Inter-partner relationships
8	Time men dedicate to childcare compared to women	Inter-partner relationships
9	Constituents of healthy relationships and sexuality; respectful communication. Excessive drinking, relationships and children. Helping partner reduce drinking	Inter-partner relationships
Mixed-sex		
10	Rationale for mixed-sex sessions. Review first 9 sessions, outline future sessions	
11	Beliefs, values and traditions in child rearing, ‘respectability’ and good behaviour. Positive discipline techniques	Reduce harsh parenting
12	Value of parental engagement in child’s education	Supporting education
13	Challenging discriminatory gender norms. Encouraging partnership in parenting	Gendered socialisation
14	Different perspectives of mothers and fathers. Resolving disputes, expressing needs and feelings, listening empathically	Inter-partner relationships
15	Participants’ experience of programme. Value of continued meetings	Reflections on programme
16	Award of certificates. Celebrations	Graduation

## Step 4: Delivering the Mechanisms of Change

Once the mechanisms of change had been clarified, it was necessary to work out how they would be delivered to the target audience. Building on the team’s prior knowledge and experience and lessons from other parenting programmes (London Economics, 2016) it was resolved that group delivery was likely to be more practical and cost-efficient than individual delivery, and more likely to modify shared social norms. The necessary steps involved in the delivery of *PfR* are set out in Table 3.

**Table 3.** Process of Delivery.

Steps		Likely duration
1	Programme’s rationale explained at local authority level and suitable villages/wards identified	1–2 weeks to arrange, 1–2 h meeting
2	Programme’s rationale explained to formal and informal community leaders and offered to the community	1–2 weeks to arrange 1–2 h meeting
3	If the programme is accepted, recruit either: a) Existing local groups, for example, religious, occupational, micro-finance or trade union, or b) ‘opinion leaders’ identified by community leaders to form parent groups.	1–2 weeks
4	Groups formed of 15–20 parents of children aged 0–17; initially single-sex	
5	Groups select two members to be trained as facilitators	
6	Facilitators attend 5 days non-residential training delivered by programme staff	1 week
7	Programme delivered to parents. Ongoing top-up training provided to facilitators intermittently over maximum 5 days	16 weeks
8	After nine sessions single-sex groups split in half and paired with those of opposite sex. Facilitators are re-paired so they too are mixed-sex	
9	Over 10 sessions groups agree steps to reduce VAC and GBV.	
10	Public graduation: Local leaders present certificates and participants testify how the programme has affected them	16th week
11	Ideally groups continue to meet with existing/new facilitators. More likely if groups single-sex, but ideally they twin with an opposite sex group for occasional meetings	Indefinite
12	Trained facilitators start new groups and recruit new facilitators	1–2 months

## Step 5: Testing and Adapting – Formative Evaluation

Although the intervention drew on the existing literature and the team’s extensive research and programming experience on gender and parent–child relationships, it was anticipated that it would require considerable refinement following initial testing. Thus, a formative evaluation was conducted of *Parenting for Good Behaviour and Respectability*, still comprising 21 sessions, from 2014 to 2016 in three stages to investigate:

1. Parents’ views of programme relevance and need to address other issues;
2. Acceptability and suitability of programme structure, content and delivery;
3. Recruitment and retention in the programme;
4. Feasibility of delivery by locally recruited facilitators; and
5. Whether it seemed to work as intended.

### Methods

#### Study Setting and Population

The study was conducted in Wakiso District, adjoining Kampala, Uganda, in two parishes `15 km apart. Kigungu is beside Lake Victoria and rural, economically reliant on fishing and small-scale farming. Bweya is peri-urban, on the Kampala–Entebbe highway, with formal and informal employment and trade as the main livelihoods. Of the 138 participants recruited, 128 continued until the end, although for some attendance was inconsistent (Table 4). In Kigungu, the programme was delivered from August 2014 to July 2015 to 47 participants in two single-sex groups which merged halfway through. This process was then repeated twice in Bweya, from May to October 2015 and February to July 2016, first with 46 and then 45 participants.

**Table 4.** Recruitment for, and Retention in, Initial PfR Groups.

	Recruited				Retained			
	Fathers	Mothers	Total no. parents	No. couples	Fathers	Mothers	Total no. parents	No. couples
Kigungu	22	25	47	7	19	23	42	7
Bweya I	17	29	46	5	14	28	42	5
Bweya II	20	25	45	4	19	25	44	4
Total	59	79	138	16	52	76	128	16

#### Data Collection

This was a descriptive study conducted pragmatically to answer the five key research questions above. Experienced Ugandan qualitative researchers, RS and FZ, used three qualitative methods. RS observed 20 sessions in each community, sampled to cover all core elements of the programme, recording implementation, participants’ responses and engagement, and issues of concern, augmented by the supervisor (CN)’s notes. Following implementation, 40 semi-structured interviews (SSIs) and three focus group discussions (FGDs) (two with mothers and one with fathers) were conducted in the respective parishes by RS and FZ in the local language, Luganda. There was generally a lengthy informal introduction to the study and the SSIs or FGDs then lasted between 1 to 2 h and were audio recorded digitally. They followed interview and FGD guides developed by the wider research team. These were slightly modified in the course of field work following data saturation on some topics and the emergence of new issues. Interviewees, aged from 19 to 63, were selected from programme participants to maximise diversity (Table 5). A FGD was conducted with each sex in Kigungu and with women in Bweya, each with six participants. They focused on participants’ impressions of the potential benefits of the programme and how to improve it.

**Table 5.** Characteristics of Interview Sample.

	Kigungu		Bweya	
	Women	Men	Women	Men
Marital status				
Married/co-habiting	7	12	6	11
Single	1	0	2	1
Livelihood				
Formal employment	2	0	0	1
Informal/self-employment	0	2	6	10
Subsistence	6	10	2	1
Age				
Over 40	3	5	2	3
Below 40	5	7	6	9
Total	8	12	8	12

#### Data Analysis

SSI and FGD recordings were transcribed verbatim and translated into English. Following familiarization with the data, a coding frame was developed by the wider team combining prior research topics and emergent issues, that is, both deductive and inductive. This was piloted with transcripts from two interviews with women, two with men, and a FGD, and revised. RS and FZ then coded the transcripts manually and summarized the coded text using framework analysis. Themes were identified by RS and FZ in consultation with the wider team, and hypotheses developed and then tested systematically against all the relevant data.

#### Ethical Issues

The study was approved by the Institutional Review Board of Makerere University and National Council for Science and Technology. Informed consent was obtained from all participants and their data pseudo-anonymized. The research team had no conflict of interest other than a wish to demonstrate the feasibility of the programme.

### Findings

#### Relevance of Programme

*Parenting for Respectability* was initially presented to community leaders as a programme to help parents bring up their children to be well behaved and respectful. This was welcomed by all communities although with limited awareness of the programme’s content. Local leaders and parents said the programme was timely since there were no formal parenting interventions in their communities.

*This project has come at the right time. Raising children in our area is a big challenge. Parents send them to school and even leave them there during holidays because they cannot manage them at home. This has created a bad relationship between parents and children because they do not interact*. Local leader, mobilization meeting, Kigungu.

*I am very grateful .... Parents (especially fathers) are always away in the lake and have very little time to be with their children for good parenting*. Local leader, mobilization meeting, Kigungu

*This is a very good programme that all of us parents need especially at such a time when parenting roles are left for house girls and teachers*. Local leader, mobilization meeting, Bweya.

Interviewed after completing the programme, participants overwhelmingly valued it, especially its focus on bringing up well behaved, respectful children, sharing parenting responsibilities between partners, and reducing inter-partner conflict. The last theme was strengthened to meet participants’ demands.

Parents of both sexes, all ages, and different educational levels agreed on the programme’s relevance. Young caregivers reported that their learning would benefit them until old age, while elderly caregivers regretted not having received it earlier. Educational level did not seem to affect interaction within groups.

*It was not bad to me because I thought, especially we the illiterates, had missed a lot. This programme was so relevant …very good from the beginning to the end*. SSI (09) mother, Bweya.

### Parenting Knowledge and Skills

Learning alternative methods of discipline was the most widely mentioned feature of the programme. Participants said they were harsh parents having learnt their parenting from their own parents and observing neighbours.

*Children used to give me headache as a single parent, so I used to get a stick and beat them with all my energy, thinking that’s disciplining a child, but now …, I don’t have any problem with them….* SSI (09) mother, Bweya

*I think, I am going to be a good father, because I did not know that while disciplining, I need to be in control of my temper so that I am realistic, while at the same time being firm and strict.* SSI father, Kigungu*.*

Women especially appreciated learning how to engage with their older children, their main challenge.

Both mothers and fathers also valued learning about children’s growth and development and were surprised by the importance of play. They reported now spending more time with their children and playing with them for the first time.

*These sessions gave us good knowledge, especially in relating with children… I did not know that playing was important for the growth of the child’s brain…* FGD (01) father, Kigungu

### Sharing Childcare Responsibilities

Both sexes found the theme of sharing household responsibilities valuable. Women welcomed it for men, and several men reported engaging more in parenting. They attributed their previous minimum input to traditional culture which classified this as women’s activity.

*But I learnt that wives are also people like us and sharing of housework would help in reducing the workload for women. The wife goes to hospital, she is sick, she comes back to wash and cook, but if she comes back and you have done some of those things for her, she becomes happy and knows that you’re the person she can depend on.* FGD (01) father, Kigungu.

Some men reported how the programme had made them aware of oppressing their women, and one explained why it was essential that men attend:

*If I had not participated, your training would have been wasted because a woman can come and tell you ‘we learnt this, handle children like this’, yet one has no idea, so it does not work. So it was good to train both a man and a woman because you both share the responsibility of parenting children.* SSI (15) father, Bweya

### Inter-Partner Conflict

Inter-partner conflict was considered very relevant, with GBV said to be widespread, especially amongst fishing communities. Participants attributed this mainly to alcoholism, adultery and poor communication skills between partners. Women said they had few options for seeking advice, but they could now share their concerns with other participants. Both sexes valued discussing marital relations and they frequently sought the facilitator’s advice after sessions.

Women asked for this theme to be expanded, partly because inter-partner conflict was thought to severely affect children. When abused by their partners, mothers could turn their anger on their children, and children suffered if they witnessed parental conflict:

*They would fight, not caring about the children… the children would cry, and the situation could get worse. The mother would cry, and the children also start crying and one could see that the children are also affected*. FGD (02) mother, Kigungu.

### Community Resistance

However, interviewees reported various negative perceptions of the programme amongst non-participants. These included that it is futile since one’s forbears did not need it, is only for the educated, would be like returning to school, is a political programme, or that parenting is irrelevant to men.

#### Acceptability of Structure, Content and Mode of Delivery

Broadly speaking the structure, content and process of *PfR* were acceptable to the participants in both communities. Parents liked all sessions because they had important, inter-linked messages: They did not want any cut and instead requested more. Women embraced the programme and nearly all attended on time, but men were often delayed by returning late from work, night fishing or social functions.

### Single-Sex/Mixed-Sex Sessions

Parents valued starting with single-sex sessions and then combining sexes. Segregated sessions allowed peer socializing, built confidence to discuss issues in relatively homogeneous groups, and facilitated subsequent participation in mixed groups. Women observed that men generally thought they knew everything so they needed to engage with new ideas on their own, in particular sharing household chores, to prepare them to discuss them in mixed groups. Likewise, men observed that single-sex sessions were good for women:

*Women have many issues they want to discuss without the presence of men but fear to say so during men’s presence. I see this method had a lot of advantages;* FGD (01) father, Kigungu

For fathers, who did not immediately identify as ‘parents’, a distinct advantage of single-sex groups was that *PfR* could be presented as a ‘fathers’ programme’, which was largely successful (Siu et al., 2017).

Both sexes reported valuing the opportunity to learn new perspectives on parenting from the opposite sex, and some men said they learnt skills from the women about handling children and families. Couples were encouraged to join the same mixed-sex group, enabling them to clarify conflicting perspectives and remind each other what they had been taught. Most appreciated this, but one man dropped out rather than attend with his wife.

### Venues and Timing

To optimize accessibility and acceptability, each group chose a convenient venue and the time for their sessions. In Kigungu, both sexes chose 10 a.m.–1 p.m. and in Bweya 2–4 p.m. Session length and programme duration were not considered problematic:

*The duration …, even the time was not a problem, because the programme was important and wide. We had to study about a child from one year to 17 years, so one cannot study it for a short time and master it.* SSI (15) father, Bweya

However, in Bweya a quarter of the men dropped out because of difficulty combining sessions with their employment.

### Home Assignments and Engagement With Non-Participants

Each session concluded with assignments to practice what had been learnt and, very importantly, engage children, partners and neighbours to reach a wider population and change community norms. Both sexes reported practicing better communication with their children and partners, supporting household activities, setting behavioural limits, and playing with their children, something only a minority had previously done. Some managed to convey *PfR* messages to a wider community:

*My neighbor’s child abused me…. The father wanted to beat the child but I refused. I told him that he did not have to beat children but to talk with them calmly. …the father eventually agreed. He talked to the boy and the boy came and apologized to me.* SSI (12) mother, Kigungu.

Others reported telling their relatives about the programme by phone.

This suggests that the programme’s lessons were acceptable within the communities. However, some participants said they found it difficult to initiate discussions on parenting with their neighbours, or faced scepticism, especially from men. Some non-participants thought it strange and childish to see parents playing with their children and some considered parenting sessions a waste of time.

#### Recruitment and Retention

##### Recruitment

The acceptability of the programme was largely confirmed by recruitment and retention figures (Table 4). In Kigungu, participants were recruited from existing savings groups, augmented from the wider community, while in Bweya there was no equivalent and so recruitment was through snowballing via community leaders. The men’s groups grew further as women persuaded their partners to join or men recruited their peers. The aim was to recruit approximately 20 participants per group.

The programme targeted any parents of children aged 0–17 years and those recruited were not especially vulnerable. They were of varied ages, marital status and ethnicities. A few had no schooling, most had attended primary school and a few secondary school. Most had informal occupations but a few were in formal employment, including teachers and nurses.

In both communities, mothers were more enthusiastic to join than fathers, primarily because fathers did not see themselves as ‘parents’ (Siu et al., 2017) and men’s work schedules often conflicted with attendance, especially in Bweya. Once a few men had attended initial sessions, they mobilized others to enroll, while some women participants persuaded their partners to join, anticipating benefits for them as a couple.

The study team tried to recruit couples, succeeding with 17 (ten in Kigungu, seven in Bweya). They valued receiving and reflecting on the same training, contributing to improved relationships between themselves and their children. Those participating alone also recognized the value of couple enrollment and recommended targeting couples:

*If possible… get the men of all the women … to come…. Because my husband can change if he learns…* FGD (03) mother, Bweya.

##### Retention

Of the 138 parents recruited, 96% of mothers and 88% fathers were retained to the end of the programme (Table 4). Attendance in the women’s sessions varied from 60 to 80% and in the men’s sessions from 45 to 65%. About a quarter of participants only attended half the sessions, about three-quarters attended over 15 sessions and very few attended all 21 sessions.

Three factors were mentioned as motivating attendance. Both sexes reported their colleagues’ continued attendance and the early impact of the programme:

*What is giving me courage is that the people who were here on the first day have all come back. Secondly, my children used to fear me…. I have changed the way I interact with my children and they have consulted me on 10 different things. Now I call them to the sitting room so that they also watch the TV with me. I used to shout at them but now I have stopped.* Observation Notes, Session 3, Men’s Group, Bweya.

Perhaps most important, each participant was given an attendance allowance of UGX 4000/session (UGX 4000 = approx. 1.2 USD). This was budgeted for refreshments, but participants requested it in cash. Women invested this in their savings or small businesses, while some men used it to support their families, demonstrating concern with family wellbeing.

Despite these motivations, retention required considerable effort by the lead facilitator and trainer (CN). Participants were reminded to attend through weekly phone calls, the ‘community megaphones’ (open-air radios) and home visits, plus parents reminded each other about the sessions.

In spite of the wide acceptance of the intervention, 10% of the women and 20% of the men did not complete it. For women, this was primarily due to their husband’s objections or to marital separation, leading them to leave the village, and for men to increased occupational demands.

#### Delivery Through Locally Recruited Facilitators

*Parenting for Respectability* was designed to be delivered by facilitators identified by participants and trained by the programme coordinator (CN). An analysis of the selection, motivations and challenges of lay facilitators highlighted the importance of interpersonal skills, motivation and parenting experience in facilitator selection (Kazemi, 2016).

The intended facilitator model seemed broadly acceptable, but this was difficult to assess since, in practice, CN delivered or co-delivered much of the programme herself, especially in Kigungu, sometimes undermining facilitators’ confidence and credibility (Kazemi, 2016). The eight Bweya facilitators (four of each sex) delivered most of the programme and parents were generally complementary, valuing their semi-participatory approach. In Kigungu, the criteria for selecting facilitators was misunderstood and the mothers group selected the person that they thought would most benefit from parent training. This was not immediately recognized by the project team, participants resented her role and after three sessions she was replaced.

Participants noted two limitations of local lay facilitators. First, they discouraged disclosure of sensitive family matters:

*…some people used to keep quiet, since… they feared to reveal secrets… We used to fear them because they were our neighbours.* FGD (03) mothers, Bweya.

The second was limited training:

*The facilitators were good [confirmatory chorus]. The problem was… you trained them in a short time. I believe if you gave them more time they could be experts [supported].* FGD (03) mothers, Bweya.

The need for extended training was also identified by the facilitators themselves (Kazemi, 2016).

#### Perceived Effectiveness

##### Family Relationships

Many participants, of both sexes, reported improved relationships with both their children and partners, widely attributed to new communication skills and greater respect.

*… we are no longer harsh to the children. We now respect each other as a couple because lack of respect was a major source of the tensions and conflict… we are able to listen to one another…. We used to fight whenever we disagreed; these days if there is an issue, I walk around and… discuss when I have calmed down.* Closing ceremony, father, Bweya.

They stated the intervention had greatly changed them and several men referred to greater involvement in parenting.

*I could spend about three months without sitting in the sitting room. Now… I sit in the sitting room and talk with my children; we even wash the motorcycle together. … I am completely a changed person and we are relating well with each other in the family.* SSI (15) father, Bweya.

##### Discipline

Many illustrations provided of the programme’s effectiveness were about discipline. An elderly woman, who recommended the programme to raise respectful children, explained

*I didn’t know I could drop the stick and just talk with the children. This was a taboo. … I saw this as impossible. But I later realized it was too much and I decided to stop because the children were very fearful of me.* SSI (06) elderly mother, Kigungu

Another argued that

*The intervention was needed… because … we did not know how to discipline children….. We thought if you are rude then children will respect you, and we did not know the difference between fear and respect. So, after the programme we realized that for someone to respect you, it depends on the way you handle them, not how they fear you. People who did not know how to… talk to their children, they now… even play with them.* FGD (03) mother, Bweya.

##### Inter-Partner Conflict

There were also claims that the sessions on inter-partner conflict and informal counselling from the facilitators had saved marital relationships.

*I was fed up and had been thinking about abandoning these children, and separating from the husband. But our facilitator told me that the problems I was facing could be discussed during the training and I would get advice from fellow parents. That is what encouraged me to attend.* FGD (03) mother, Bweya.

Both sexes said increased communication with their spouses and children had reduced fighting and facilitated conflict resolution.

*…now we know that if someone does something wrong, you find a way to resolve it by staying calm… rather than taking drastic action*. SSI (03) father, Kigungu

Some Kigungu men who had left their spouses claimed that the programme enabled them to reunite, and some local leaders welcomed the impact of *PfR* on their communities:

*We were in great need of this knowledge. Women used to go to local leaders with many complaints [about domestic violence] but these days they have greatly reduced, even fighting in the homes in this village have reduced*. Closing ceremony, local leader, Bweya.

In one of the women’s FGDs, however, some women were sceptical that they could ever reverse years of rudeness and resentment towards their partners, or thought it would take a very long time.

#### Continuation Beyond Formal Programme

Having completed the programme, parents promised to continue implementing what they had learnt, so as to become role models in the community. They recommended that the programme be rolled out to others, including their adult children:

*I think that the programme should be expanded to reach other people. It was a good programme and has ended when we still want to participate… The programme should also be used to teach the older children about discipline.’* SSI (04) mother, Kigungu

Participants also promised to spread the message to other community members and, in Kigungu, to do this through their savings groups.

### Revisions to the Programme

The formative evaluation led to several revisions. Sessions were made shorter, more practical and better illustrated. The programme was reduced from 21 to 16 sessions to maximise retention and reduce costs. Although the optimal length of parenting programmes is unclear, a recent meta-regression analysis of programmes targeting child maltreatment found that programmes under 12 sessions were more effective than longer ones (Chen & Chan, 2015). This programme exceeds this due to additional content on reducing inter-partner conflict, which was given more prominence in the revised version. Finally, the working title *Parenting for Good Behaviour and Respectability* was shortened to *Parenting for Respectability (PfR).*

## Discussion and Applications to Practice

In response to the perceived need for early prevention of GBV, a community-based parenting programme was developed to modify four familial predictors of GBV: Poor child attachment and parental bonding, harsh parenting, inequitable gendered socialization and parental conflict. Its design followed the 6SQuID model of intervention development (Wight et al., 2016), the first five steps being described in this article. *Parenting for Respectability* was based on an explicit programme theory that incorporated Attachment Theory, the concept that positive behavioural control develops emotional control, and Social Learning Theory.

The formative evaluation of *PfR* in two localities with 138 participants suggests that the structure, content and mode of delivery were broadly acceptable in both. Practitioners working to prevent GBV or VAC might want to apply some of the five features of this intervention which differentiate it from most parenting programmes:

1. Focussing on the concern of both fathers and mothers that their children should be well behaved and maintain the family’s respectability;
2. Deliberately aiming to include fathers as much as mothers, starting with single-sex sessions;
3. Targeting all parents and carers of children aged from 0 to 17;
4. Promoting parents’ confidence in positive parenting and reducing partner conflict by engaging them in identifying their own solutions; and
5. Promoting healthy spousal relationships through reflection on social norms and communication.

In LICs, many programmes are dependent on locally recruited lay facilitators for widespread delivery. This formative evaluation suggests several practical lessons regarding such facilitators. Although they were generally considered suitable, the fact that, in practice, the trainer delivered much of the programme herself, indicates that even 5 days training may be inadequate to give lay facilitators the confidence and competence to deliver conceptually complex materials. It might also indicate that local facilitators were not fully acceptable, for instance they restricted disclosure of sensitive family matters. A complementary study identified facilitators’ motivations and challenges at the individual, community and organizational levels (Kazemi, 2016). Motivational factors included personal transformation and improved relationships; refresher training; modest payment; perceived changes in the community; supervision, mentoring and the inspirational content. Challenges included Self-doubt, competing obligations and inadequate remuneration; abstract text in the manual; and uncertainty around timing and expectations. The study concluded that the success of lay facilitator-led parenting programmes depends on careful community-based selection, on the basis of interpersonal skills, desire to learn and parenting experience, comprehensive training and subsequent support. However, further research is needed to identify how best to refine facilitator selection, recruitment, training and supervision.

Although the formative evaluation did not focus on outcomes, numerous participants testified how their family relationships had been changed by the programme. As intended, the programme seemed to tap into participants’ internal motivation to bring up respectful, and respectable, children, making it culturally compelling (Panter-Brick et al., 2006).

The formative evaluation allows us to go some way towards answering the key questions of Realist Evaluation (Pawson & Tilley, 1997) (Table 6).

**Table 6.** Realist Evaluation.

Question	Provisional Answer
What worked?	Delivery to single-sex and then mixed-sex groups, appreciative enquiry, learning positive discipline and conflict resolution skills, local venues and an attendance allowance
For whom?	Participants were self-selecting: It is unclear whether they represented the most or least dis-functional families, or were typical. Women engaged more readily than men, but seven-eighths of men remained to the end. Their high level of involvement contrasts with most parenting programmes Stahlschmidt et al. (2013) and is probably due to: starting the programme in single-sex groups; exploiting men’s concern with family respectability; and the participatory style Siu et al. (2017). *PfR* was least appropriate for men in formal employment, making it more suitable for rural than peri-urban/urban locations
In what circumstances?	Where parents were concerned about their children’s behaviour and family respectability, there was a high prevalence of GBV, parents were not constrained by formal employment, there had been no previous parenting programmes and none providing large incentives for participation. In so far as *PfR* influenced the wider geographical community beyond direct participants, this will depend on the proportion of community members participating and the density of community networks
Why?	The target group considered the programme highly relevant to their lives and were strongly motivated to improve their children’s behaviour, share parenting responsibilities between partners, and resolve marital conflict. The last element was strengthened in response to demand. Perceived evidence of effectiveness encouraged participation

There are several limitations to this study. The formative evaluation was not designed to provide evidence of effectiveness and only generated unsystematic, qualitative, self-reported data on the impact of the programme. Although this was overwhelmingly positive, it is well established that the validity of such accounts is highly problematic, especially when provided to researchers associated with the programme. Furthermore, we should be transparent in noting that, apart from FZ, all those involved in collecting data and analysing them had been directly involved in designing the programme and were therefore likely to have had a positive bias in interpreting them (Levitt et al., 2018).

The research findings were suggestive, not definitive, about which components of the intervention were most valuable and which might be condensed or cut. Ideally this might be resolved through a factorial experiment (Collins, 2018). Nor could the research identify the optimum choice of facilitators or the optimum training and support needed, given resource constraints. Data were not collected on participants’ family size or income level, preventing analysis of how these might have affected engagement with the programme.

The programme was only tested in two villages within one district in Uganda, with an overwhelmingly Baganda population. It cannot be assumed that these findings can be generalized to other ethnic groups or geographical areas. The occupational diversity of the sample improves generalizability, but the programme would still need to be tested with predominantly pastoralist populations. Nearly all those interviewed participated in almost the entire programme, therefore excluding those who dropped out or initially declined to participate. Such people are likely to provide important perspectives on barriers to participation and engagement (Watters & Biernacki, 1989). Finally, the formative evaluation did not focus on how children, the intended main beneficiaries of the intervention, experienced their parents’ involvement in it. Research with them might have identified important ways to improve the programme, for example, through involving children directly, or important unintended consequences. Subsequent research has shown that children are not as concerned about corporal punishment as are its critics (Bates, 2018).

Notwithstanding these limitations, the careful development of this community-based parenting programme over 3 years has resulted in a promising programme for the early prevention of GBV. An extensive formative evaluation identified several strengths of *PfR*, in particular its cultural appropriateness, and various ways to improve it, although uncertainties about the optimum mode of delivery still remain. The sixth step of the SQuID model, to establish initial evidence of effectiveness, was subsequently pursued through a pre-post evaluation with 484 parents. Ugandan NGOs and local authority community development officers are keen to implement the programme widely, but its effectiveness is still to be confirmed through a randomized controlled trial, currently underway.

## References

[bibr1-10497315211056246] Abrahams N. Jewkes R. (2005). Effects of South African men’s having witnessed abuse of their mothers during childhood on their levels of violence in adulthood. American Journal of Public Health, 95(10), 1811–1816. https://doi.org./10.2105/AJPH.2003.035006 16131646 10.2105/AJPH.2003.035006 PMC1449441

[bibr2-10497315211056246] Abramsky T. Devries K.M. Michau L. Nakuti J. Musuya T. Kyegombe N. Watts C. (2016). The impact of SASA!, a community mobilisation intervention, on women’s experiences of intimate partner violence: Secondary findings from a cluster randomised trial in Kampala, Uganda. Journal of Epidemiology and Community Health, 70(8), 818–825. https://doi.org./10.1136/jech-2015-206665 26873948 10.1136/jech-2015-206665 PMC4975800

[bibr3-10497315211056246] Ainsworth M. S. (1967). Infancy in Uganda. Infant care and the growth of love. The John’s Hopkins Press.

[bibr4-10497315211056246] Ainsworth M. Blehar M. Waters E. Wall S. (1978). Patterns of Attachment: A Psychological Study of the Strange Situation. Erlbaum.

[bibr5-10497315211056246] Anda R. F. Butchart A. Felitti V. J. Brown D. W. (2010). Building a framework for global surveillance of the public health implications of adverse childhood experiences. American Journal of Preventive Medicine, 39(1), 93–98. 20547282 10.1016/j.amepre.2010.03.015

[bibr6-10497315211056246] Ashburn K. Kerner B. Ojamuge D. Lundgren R. (2017). Evaluation of the responsible, engaged, and loving (REAL) fathers initiative on physical child punishment and intimate partner violence in Northern Uganda. Prevention Science, 18(7), 854–864. 27738782 10.1007/s11121-016-0713-9 PMC5602091

[bibr7-10497315211056246] Bacchus L. J. Colombini M. Urbina M. C. Howarth E. L. Gardner F. Annan J. Ashburn K. Madrid B. Levtov R. Watts C. (2017). Exploring opportunities for coordinated responses to intimate partner violence and child maltreatment in low and middle income countries: A scoping review. Psychology, Health & Medicine, 22(sup1), 135–165. 10.1080/13548506.2016.1274410 28150500

[bibr8-10497315211056246] Bandura A. (1986). Social foundations of thought and action: A social cognitive theory. Prentice-Hall.

[bibr9-10497315211056246] Bates R. (2018). Children’s experiences of, and perspectives on, their relationships with their parents: A qualitative study in Wakiso district, Central Uganda [Unpublished master’s thesis, University of Glasgow].

[bibr10-10497315211056246] Bavolek S. (1989). Research and validation of the adult adolescent parenting inventory (AAPI). Family Development Resources.

[bibr11-10497315211056246] Belsky J. Fearon R. M. (2008). Precursors of attachment security. In J Cassidy P Shaver (Eds.), Handbook of attachment (2nd ed., pp. 295–316). Guilford Press.

[bibr12-10497315211056246] Bitel M. (2012). Evidence-based lessons learned from evaluations on community participation and civil society involvement in health EvalBrief oct., No. 2. Swiss Agency for Development and Cooperation SDC.

[bibr13-10497315211056246] Bostrom M. (2003). Discipline and development: A meta-analysis of public perceptions of parents, parenting, child development and child abuse. Frameworks Institute.

[bibr14-10497315211056246] Bowlby J. (1982). Attachment. Basic Books.

[bibr15-10497315211056246] Bronfenbrenner U. (1979). The ecology of human development: Experiments by nature and design. Harvard University Press.

[bibr16-10497315211056246] Brown J. Cohen P. Johnson J. G. Salzinger S. (1998). A longitudinal analysis of risk factors for child maltreatment: Findings of a 17-year prospective study of officially recorded and self-reported child abuse and neglect. Child Abuse & Neglect, 22(11), 1065–1078. 9827312 10.1016/s0145-2134(98)00087-8

[bibr17-10497315211056246] Bunton R. Murphy S. Bennett P. (1991). Theories of behavioural change and their use in health promotion: Some neglected areas. Health Education Research, 6(2), 153–162.

[bibr18-10497315211056246] Butchart A. Harvey A. P. Mian M. Furniss T. (2006). Preventing child maltreatment: A guide to taking action and generating evidence. World Health Organization and International Society for Prevention of Child Abuse and Neglect.

[bibr19-10497315211056246] Capaldi D. M. Clark S. (1998). Prospective family predictors of aggression toward female partners for at-risk young men. Developmental Psychology, 34(6), 1175–1188. 9823503 10.1037//0012-1649.34.6.1175

[bibr20-10497315211056246] Chen M. Chan K. L. (2015). Effects of parenting programs on child maltreatment prevention: A meta-analysis. Trauma, Violence & Abuse, 17(1), 88–104. 10.1177/1524838014566718 25573846

[bibr21-10497315211056246] Collins L. M. (2018). Optimization of behavioral, biobehavioral, and biomedical interventions: The multiphase optimization strategy (MOST). Springer International Publishing.

[bibr22-10497315211056246] Connell R. W. (1987). Gender and power. Stanford University Press.

[bibr23-10497315211056246] Cooper P. J. Tomlinson M. Swartz L. Landman M. Molteno C. Stein A. McPherson K. Murray L. (2009). Improving quality of mother-infant relationship and infant attachment in socioeconomically deprived community in South Africa: Randomised controlled trial. British Medical Journal, 338, b974. 19366752 10.1136/bmj.b974 PMC2669116

[bibr24-10497315211056246] Cork C. White R. Noel P. Bergin N. (2018). Randomized controlled trials of interventions addressing intimate partner violence in Sub-Saharan Africa: A systematic review. Trauma, Violence & Abuse, 21(4), 643–659. 10.1177/1524838018784585 PMC7197024 29962286

[bibr25-10497315211056246] Cornwall A. (2009). Changing ideals in a donor organisation: ‘Participation’in Sida. IDS Editorial Team, 2009(317), 1–31).

[bibr26-10497315211056246] Danese A. McEwen B. S. (2012). Adverse childhood experiences, allostasis, allostatic load, and age-related disease. Physiology & Behavior, 106(1), 29–39. 21888923 10.1016/j.physbeh.2011.08.019

[bibr27-10497315211056246] Devlin A. M. Wight D. Fenton C. (2018). Are parenting practices associated with the same child outcomes in sub-Saharan African countries as in high-income countries? A review and synthesis. BMJ Global Health, 3(6), e000912. 10.1136/bmjgh-2018-000912 PMC6326425 30687520

[bibr28-10497315211056246] Devries K. M. Child J. C. Allen E. Walakira E. Parkes J. Naker D. (2014). School violence, mental health, and educational performance in Uganda. Pediatrics, 133(1), e129–e137. 24298004 10.1542/peds.2013-2007

[bibr29-10497315211056246] Djeddah C. Facchin P. Ranzato C. Romer C. (2000). Child abuse: Current problems and key public health challenges. Social Science & Medicine, 51(6), 905–915. 10972434 10.1016/s0277-9536(00)00070-8

[bibr30-10497315211056246] Dutton D. G. (1999). Traumatic origins of intimate rage. Aggression and Violent Behavior, 4(4), 431–447.

[bibr31-10497315211056246] Ellsberg M. Arango D. J. Morton M. Gennari F. Kiplesund S. Contreras M. Watts C. (2015). Prevention of violence against women and girls: What does the evidence say? Lancet(9977), 385, 1555–1566. 25467575 10.1016/S0140-6736(14)61703-7

[bibr32-10497315211056246] Engle P. L. Fernald L. C. Alderman H. Behrman J. O'Gara C. Yousafzai A. De Mello M. C. Hidrobo M. Ulkuer N. Ertem I. Iltus S. Global Child Development Steering Group (2011). Strategies for reducing inequalities and improving developmental outcomes for young children in low-income and middle-income countries. Lancet, 378(9799), 1339–1353. 21944378 10.1016/S0140-6736(11)60889-1

[bibr34-10497315211056246] Farrington D. P. (1998). Predictors, causes, and correlates of male youth violence. In M Tonry MH Moore (Eds.), Youth violence (pp. 421–475). University of Chicago Press.

[bibr35-10497315211056246] Felitti V. J. Anda R. F. Nordenberg D. Williamson D. F. Spitz A. M. Edwards V. Marks J. S. (1998). Relationship of childhood abuse and household dysfunction to many of the leading causes of death in adults: The adverse childhood experiences (ACE) study. American Journal of Preventive Medicine, 14(4), 245–258. 9635069 10.1016/s0749-3797(98)00017-8

[bibr36-10497315211056246] Fulu E. Jewkes R. Roselli T. Garcia-Moreno C. (2013). Prevalence of and factors associated with male perpetration of intimate partner violence: Findings from the UN multi-country cross-sectional study on men and violence in Asia and the Pacific. The Lancet Global Health, 1(4), e187–e207. 25104345 10.1016/S2214-109X(13)70074-3

[bibr37-10497315211056246] Gardner F. E. (1989). Inconsistent parenting: Is there evidence for a link with children’s conduct problems? Journal of Abnormal Child Psychology, 17(2), 223–233. 2745902 10.1007/BF00913796

[bibr38-10497315211056246] Gershoff E. T. (2002). Corporal punishment by parents and associated child behaviors and experiences: A meta-analytic and theoretical review. Psychological Bulletin, 128(4), 539–579. 12081081 10.1037/0033-2909.128.4.539

[bibr39-10497315211056246] Glaser D. (2000). Child abuse and neglect and the brain: A review. Journal of Child Psychology and Psychiatry, 41(1), 97–116. 10763678

[bibr40-10497315211056246] Global Initiative to End All Corporal Punishment of Children (2017). Corporal punishment of children in Uganda. https://endcorporalpunishment.org/reports-on-every-state-and-territory/uganda/.

[bibr41-10497315211056246] Grusec J. E. Goodnow J. J. (1994). Impact of parental discipline methods on the child’s internalization of values: A reconceptualization of current points of view. Developmental Psychology, 30(1), 4–19.

[bibr42-10497315211056246] Gubi D. Nansubuga E. Wandera S.O. (2020). Correlates of intimate partner violence among married women in Uganda: A cross-sectional survey. BMC Public Health, 20(1), 1008. 10.1186/s12889-020-09123-4 32586297 PMC7318470

[bibr43-10497315211056246] Guedes A. Bott S. Garcia-Moreno C. Colombini M. (2016). Bridging the gaps: A global review of intersections of violence against women and violence against children. Global Health Action, 9(1), 31516. 10.3402/gha.v9.31516 27329936 PMC4916258

[bibr44-10497315211056246] Harvey A. Garcia-Moreno C. Butchart A. (2007). Primary prevention of intimate-partner violence and sexual violence: Background. paper for WHO expert meeting May 2–3, 2007. World Health Organization, Department of Violence and Injury Prevention and Disability.

[bibr45-10497315211056246] Hatcher A. Colvin C. J. Ndlovu N. Dworkin S. L. (2014). Intimate partner violence among rural South African men: Alcohol use, sexual decision-making and partner communication. Culture, Health & Sexuality, 16(9), 1023–1039. 10.1080/13691058.2014.924558 PMC4490163 24939358

[bibr46-10497315211056246] Hawkins J. D. Herrenkohl T. Farrington D. P. Brewer D. Catalano R. F. Harachi T. W. (1998). A review of predictors of youth violence. In DP Farrington R Loeber (Eds.), Serious and violent juvenile offenders: Risk factors and successful interventions. Sage.

[bibr47-10497315211056246] Heise L. (2011). What works to prevent partner violence? An evidence overview. Strive Research Consortium.

[bibr48-10497315211056246] Heise L. Ellsberg M. Gottmoeller M. (2002). A global overview of gender‐based violence. International Journal of Gynecology & Obstetrics, 78(Suppl 1), S5–S14. 12429433 10.1016/S0020-7292(02)00038-3

[bibr49-10497315211056246] Herrenkohl T. I. Herrenkohl R. C. (2007). Examining the overlap and prediction of multiple forms of child maltreatment, stressors, and socioeconomic status: A longitudinal analysis of youth outcomes. Journal of Family Violence, 22(7), 553–562.

[bibr50-10497315211056246] Hillis S. D. Anda R. F. Felitti V. J. Nordenberg D. Marchbanks P. A. (2000). Adverse childhood experiences and sexually transmitted diseases in men and women: A retrospective study. Pediatrics, 106(1), E11. 10878180 10.1542/peds.106.1.e11

[bibr51-10497315211056246] Hillis S. Mercy J. Amobi A. Kress H. (2016). Global prevalence of past-year violence against children: A systematic review and minimum estimates. Pediatrics, 137(3), e20154079. 26810785 10.1542/peds.2015-4079 PMC6496958

[bibr52-10497315211056246] Hoffmann T. C. Glasziou P. P. Boutron I. Milne R. Perera R. Moher D. Altman D. G. Barbour V. Macdonald H. Johnston M. Lamb S. E. Dixon-Woods M. McCulloch P. Wyatt J. C. Chan A. Michie S. (2014). Better reporting of interventions: Template for intervention description and replication (TIDieR) checklist and guide. British Medical Journal, 348, g1687. 24609605 10.1136/bmj.g1687

[bibr53-10497315211056246] Holt S. Buckley H. Whelan S. (2008). The impact of exposure to domestic violence on children and young people: A review of the literature. Child Abuse & Neglect, 32(8), 797–810. 18752848 10.1016/j.chiabu.2008.02.004

[bibr54-10497315211056246] Hutchings J. Gardner F. (2012). Support from the start: Effective programmes for three-eight-year-olds. Journal of Children’s Services, 7(1), 29–40.

[bibr55-10497315211056246] Jewkes R. (2002). Intimate partner violence: Causes and prevention. Lancet, 359(9315), 1423–1429. 11978358 10.1016/S0140-6736(02)08357-5

[bibr56-10497315211056246] Jewkes R. K. Dunkle K. Nduna M. Shai N. (2010). Intimate partner violence, relationship power inequity, and incidence of HIV infection in young women in South Africa: A cohort study. Lancet, 376(9734), 41–48. 20557928 10.1016/S0140-6736(10)60548-X

[bibr57-10497315211056246] Kazemi M. (2016). Motives and challenges of lay facilitators implementing a parenting program to reduce gender-based violence in Wakiso district, Uganda [Unpublished masters dissertation, Global Health London School of Hygiene and Tropical Medicine].

[bibr59-10497315211056246] Knerr W. Gardner F. Cluver L. (2013). Improving positive parenting skills and reducing harsh and abusive parenting in low-and middle-income countries: A systematic review. Prevention Science, 14(4), 352–363. 23315023 10.1007/s11121-012-0314-1

[bibr60-10497315211056246] Lachman J. M. Kelly J. Cluver L. Ward C. L. Hutchings J. Gardner F. (2018). Process evaluation of a parenting program for low-income families in South Africa. Research on Social Work Practice, 28(2), 188–202.

[bibr61-10497315211056246] Lachman J. Wamoyi J. Spreckelsen T. Wight D. Maganga J. Gardner F. (2020). Combining parenting and economic strengthening programmes to reduce violence against children: A cluster randomised controlled trial with predominantly male caregivers in rural Tanzania. BMJ Global Health, 5(7), e002349. 10.1136/bmjgh-2020-002349 PMC7348478 32641291

[bibr62-10497315211056246] Lansford J. E. Chang L. Dodge K. A. Malone P. S. Oburu P. Palmérus K. Bacchini D. Pastorelli C. Bombi A. S. Zelli A. Tapanya S. Chaudhary N. Deater-Deckard K. Manke B. Quinn N. (2005). Physical discipline and children’s adjustment: Cultural normativeness as a moderator. Child Development, 76(6), 1234–1246. 16274437 10.1111/j.1467-8624.2005.00847.x PMC2766084

[bibr63-10497315211056246] Lansford J. E. Deater‐Deckard K. (2012). Childrearing discipline and violence in developing countries. Child Development, 83(1), 62–75. 22277007 10.1111/j.1467-8624.2011.01676.x

[bibr64-10497315211056246] Levitt H. M. Creswell J. W. Josselson R. Bamberg M. Frost D. M. Sarrez-Orozco C. (2018). Journal article reporting standards for qualitative primary, qualitative meta-analytic, and mixed methods research in psychology: The APA publications and communications board task force report. American Psychologist, 73(1), 26–46. 10.1037/amp0000151 29345485

[bibr65-10497315211056246] Lichter E. L. McCloskey L. A. (2004). The effects of childhood exposure to marital violence on adolescent gender-role beliefs and dating violence. Psychology of Women Quarterly, 28(4), 344–357.

[bibr66-10497315211056246] London Economics (2016). Cost benefit analysis of interventions with parents. (Research report DCSF-RW008). Dept. for Children, Schools and Families.

[bibr68-10497315211056246] Maas C. Herrenkohl T. I. Sousa C. (2008). Review of research on child maltreatment and violence in youth. Trauma, Violence & Abuse, 9(1), 56–67. 10.1177/1524838007311105 18182631

[bibr69-10497315211056246] Main M. Solomon J. (1990). Procedures for identifying infants as disorganized/disoriented during the ainsworth strange situation. In M Greenberg D Cicchetti E Cummings (Eds.), Attachment in the preschool years: Theory, research and intervention (pp. 121–160). University of Chicago Press.

[bibr71-10497315211056246] McKee L. Roland E. Coffelt N. Olson A. L. Forehand R. Massari C. Jones D. Gaffney C. A. Zens M. S. (2007). Harsh discipline and child problem behaviors: The roles of positive parenting and gender. Journal of Family Violence, 22(4), 187–196.

[bibr72-10497315211056246] McLeroy K. R. Bibeau D. Steckler A. Glanz K. (1988). An ecological perspective on health promotion programs. Health Education Quarterly, 15(4), 351–377. 3068205 10.1177/109019818801500401

[bibr73-10497315211056246] MGLSD (2017). Uganda violence against children survey: Findings from a National Survey 2015. Ministry of Gender, Labour and Social Development.

[bibr74-10497315211056246] Michie S. Van Stralen M. M. West R. (2011). The behaviour change wheel: A new method for characterising and designing behaviour change interventions. Implementation Science, 6(1), 1–12. 10.1186/1748-5908-6-42 21513547 PMC3096582

[bibr75-10497315211056246] Morelli G. Quinn N. Chaudhary N. Vicedo M. Rosabal-Coto M. Keller H. Murray M. Gottlieb A. Scheidecker G. Takada A. (2018). Ethical challenges of parenting interventions in low-to middle-income countries. Journal of Cross-Cultural Psychology, 49(1), 5–24.

[bibr76-10497315211056246] Murray J. Anselmi L. Gallo E. A. G. Fleitlich-Bilyk B. Bordin I. A. (2013). Epidemiology of childhood conduct problems in Brazil: Systematic review and meta-analysis. Social Psychiatry and Psychiatric Epidemiology, 48(10), 1527–1538. 23644723 10.1007/s00127-013-0695-x PMC3782642

[bibr77-10497315211056246] Murray J. Farrington D. P. (2010). Risk factors for conduct disorder and delinquency: Key findings from longitudinal studies. The Canadian Journal of Psychiatry, 55(10), 633–642. 20964942 10.1177/070674371005501003

[bibr78-10497315211056246] Norton R. Kobusingye O. (2013). Injuries. The New England Journal of Medicine, 368(18), 1723–1730. 23635052 10.1056/NEJMra1109343

[bibr79-10497315211056246] Panter-Brick C. Clarke S. E. Lomas H. Pinder M. Lindsay S. W. (2006). Culturally compelling strategies for behaviour change: A social ecology model and case study in malaria prevention. Social Science & Medicine, 62(11), 2810–2825. 16352385 10.1016/j.socscimed.2005.10.009

[bibr80-10497315211056246] Pawson R. Tilley N. (1997). Realistic evaluation. Sage.

[bibr82-10497315211056246] Population Council (2008). Sexual and gender based violence in Africa. Literature review. Population Council.

[bibr85-10497315211056246] Rothbaum F. Weisz J. R. (1994). Parental caregiving and child externalizing behavior in nonclinical samples: A meta-analysis. Psychological Bulletin, 116(1), 55–74. 8078975 10.1037/0033-2909.116.1.55

[bibr86-10497315211056246] Schwartz J. P. Hage S. M. Bush I. Burns L. K. (2006). Unhealthy parenting and potential mediators as contributing factors to future intimate violence: A review of the literature. Trauma, Violence & Abuse, 7(3), 206–221. 10.1177/1524838006288932 16785287

[bibr87-10497315211056246] Seedat M. Van Niekerk A. Jewkes R. Suffla S. Ratele K. (2009). Violence and injuries in South Africa: Prioritising an agenda for prevention. Lancet, 374(9694), 1011–1022. 19709732 10.1016/S0140-6736(09)60948-X

[bibr88-10497315211056246] Shenderovich Y. Eisner M. Cluver C. Doubt J. Berezin M. Majokweni S. Murray A. L. (2019). Delivering a parenting program in South Africa: The impact of implementation on outcomes. Journal of Child and Family Studies, 28(4), 1005–1017.

[bibr89-10497315211056246] Singla D. R. Kumbakumba E. Aboud F. E. (2015). Effects of a parenting intervention to address maternal psychological wellbeing and child development and growth in rural Uganda: A community-based, cluster-randomised trial. The Lancet Global Health, 3(8), e458–e469. 26144389 10.1016/S2214-109X(15)00099-6

[bibr90-10497315211056246] Siu G. E. Seeley J. Wight D. (2013). Dividuality, masculine respectability and reputation: How masculinity affects men’s uptake of HIV treatment in rural eastern Uganda. Social Science & Medicine, 89, 45–52. 10.1016/j.socscimed.2013.04.025. 23726215

[bibr91-10497315211056246] Siu G. E. Wight D. Seeley J. Namutebi C. Sekiwunga R. Zalwango F. Kasule S. (2017). Men’s involvement in a parenting programme to reduce child maltreatment and gender-based violence: Formative evaluation in Uganda. The European Journal of Development Research, 29(5), 1017–1037.

[bibr92-10497315211056246] Smith C. A. Stern S. B. (1997). Delinquency and antisocial behavior: A review of family processes and intervention research. Social Service Review, 71(3), 382–420.

[bibr93-10497315211056246] Stahlschmidt M. J. Threlfall J. Seay K. D. Lewis E. M. Kohl P. L. (2013). Recruiting fathers to parenting programs: Advice from dads and fatherhood program providers. Children and Youth Services Review, 35(10), 1734–1741. 24791035 10.1016/j.childyouth.2013.07.004 PMC4002003

[bibr95-10497315211056246] Straus M. A. Douglas E. M. Medeiros R. A. (2013). The primordial violence: Spanking children, psychological development, violence, and crime. Routledge.

[bibr97-10497315211056246] Trentacosta C. J. Hyde L. W. Shaw D. S. Dishion T. J. Gardner F. Wilson M. (2008). The relations among cumulative risk, parenting, and behavior problems during early childhood. Journal of Child Psychology and Psychiatry, 49(11), 1211–1219. 18665880 10.1111/j.1469-7610.2008.01941.x PMC2683369

[bibr98-10497315211056246] Uganda Bureau of Statistics and ICF (2018). Uganda demographic and health survey 2016. UBOS and ICF.

[bibr99-10497315211056246] Wamoyi J. Wight D. (2014). “Dying a hero:” Parents’ and young people’s discourses on concurrent sexual partnerships in rural Tanzania. BMC Public Health, 14(1), 742. 25048413 10.1186/1471-2458-14-742 PMC4223426

[bibr100-10497315211056246] Warrington M. (2013). Challenging the status quo: The enabling role of gender sensitive fathers, inspirational mothers and surrogate parents in Uganda. Educational Review, 65(4), 402–415.

[bibr101-10497315211056246] Watters J. K. Biernacki P. (1989). Targeted sampling: Options for the study of hidden populations. Social Problems, 36(4), 416–430.

[bibr103-10497315211056246] Webster-Stratton C. (1998). Preventing conduct problems in Head Start children: Strengthening parenting competencies. Journal of Consulting and Clinical Psychology, 66(5), 715–730. 9803690 10.1037//0022-006x.66.5.715

[bibr104-10497315211056246] Welbourn A. Kilonzo F. Mboya T. J. Liban S. M. (2016). Stepping stones and stepping stones plus: A training package on gender, generation, HIV, communication and relationship skills. Practical Action Publishing.

[bibr105-10497315211056246] Wight D. Remes P. (2010). Addressing parenting in HIV prevention—lessons from Mema kwa Jamii. [Conference presentation]. Countdown to 2015: Challenging orthodoxies related to SRH and HIV. DfID conference, London, UK, 17–18 May 2015. London South Bank University.

[bibr106-10497315211056246] Wight D. Wimbush E. Jepson R. Doi L. (2016). Six steps in quality intervention development (6SQuID). Journal of Epidemiology and Community Health, 70(5), 520–525. 26573236 10.1136/jech-2015-205952 PMC4853546

[bibr107-10497315211056246] Wood K. Lambert H. Jewkes R. (2008). “Injuries are beyond love:” Physical violence in young South Africans’ sexual relationships. Medical Anthropology, 27(1), 43–69. 18266171 10.1080/01459740701831427

[bibr108-10497315211056246] World Health Organization (2006). Mental health and psychosocial well–being among children in severe food shortage situations. Department of Mental Health and Substance AbuseWorld Health Organization.

[bibr109-10497315211056246] World Health Organization (2007). Helping parents in developing countries improve adolescents’ health. World Health Organization.

[bibr110-10497315211056246] World Health Organization (2013). Preventing violence: Evaluating outcomes of parenting programmes. World Health Organization.

[bibr111-10497315211056246] World Health Organization (2021 2018). Violence against women prevalence estimates. World Health Organization.

[bibr112-10497315211056246] World Health Organization/London School of Hygiene and Tropical Medicine (2010). Preventing intimate partner and sexual violence against women: Taking action and generating evidence. World Health Organization.

